# Assessing Antioxidant Capacity in Brain Tissue: Methodologies and Limitations in Neuroprotective Strategies

**DOI:** 10.3390/antiox3040636

**Published:** 2014-10-13

**Authors:** Jennifer E. Slemmer, John T. Weber

**Affiliations:** 1 Bioscience Technology, Holland College, 140 Weymouth Street, Charlottetown, PE, C1A 4Z1, Canada; E-Mail: jeslemmer@hollandcollege.com; 2 School of Pharmacy, Memorial University of Newfoundland, 300 Prince Philip Drive, St. John’s, NL, A1B 3V6, Canada

**Keywords:** antioxidant, bioavailability, cell culture, neuroprotection

## Abstract

The number of putative neuroprotective compounds with antioxidant activity described in the literature continues to grow. Although these compounds are validated using a variety of *in vivo* and *in vitro* techniques, they are often evaluated initially using *in vitro* cell culture techniques in order to establish toxicity and effective concentrations. Both *in vivo* and *in vitro* methodologies have their respective advantages and disadvantages, including, but not limited to, cost, time, use of resources and technical limitations. This review expands on the inherent benefits and drawbacks of *in vitro* and *in vivo* methods for assessing neuroprotection, especially in light of proper evaluation of compound efficacy and neural bioavailability. For example, *in vivo* studies can better evaluate the effects of protective compounds and/or its metabolites on various tissues, including the brain, in the whole animal, whereas *in vitro* studies can better discern the cellular and/or mechanistic effects of compounds. In particular, we aim to address the question of appropriate and accurate extrapolation of findings from *in vitro* experiment-where compounds are often directly applied to cellular extracts, potentially at higher concentrations than would ever cross the blood-brain barrier—to the more complex scenario of neuroprotection due to pharmacodynamics *in vivo*.

## 1. Introduction

The present review focuses on the relative advantages, disadvantages, and methodological concerns when assessing antioxidant compounds for the treatment of various health conditions, particularly related to the nervous system. Our analysis of the literature, including our own work using neuroprotective compounds, indicates that extrapolating findings from *in vitro* experiments to neuroprotection *in vivo* requires appropriate methodological choices, as well as recognition of the limitations of *in vitro* experiments. Some of these methodologies involve examining the structure-function relationships of successful neuroprotective compounds; for this reason, we describe some specific molecules, such as various polyphenols, which have been successful *in vivo*, in both rodents and humans, in enhancing cognitive function, and compare them to some molecules that possessed promise on paper, but did not perform well in human clinical trials.

Initial findings and studies are almost always conducted using *in vitro* techniques, with either cell lines or primary cell cultures, before moving successful compounds into the whole animal. As described in detail in future sections, the use of *in vitro* experimentation is a faster, more cost-effective manner to screen compounds from a diverse panel of candidates. Many of the compounds that we describe in this review are antioxidants, and for this reason, we focus on the effects of oxidative stress on brain tissue, and the treatment of various conditions, such as stroke, with antioxidant compounds. Again, the choice of methodology is critical when investigating a compound’s neuroprotective capacity. Is the compound a true antioxidant, in that it is capable of neutralizing oxidizing molecules that could damage cell membranes and/or DNA, or does it have a different mechanism of action? If the experimental design does not allow for several possibilities, then a potentially beneficial compound may be rejected for failing to provide direct neuronal protection *in vitro*. Our aim is to shed light on the relative advantages and disadvantages of *in vitro* and *in vivo* methodologies for assessing potential neuroprotective, antioxidant compounds, and to provide guidance for studies using these compounds in the future.

## 2. Cellular Mechanisms of Protection to the Brain Due to Antioxidant Activity

### 2.1. Redox Homeostasis and Oxidative Stress

Oxidative stress has been purported to be a common underlying mechanism behind many human health conditions, such as stroke, traumatic brain injury, and aging [1]. Briefly, oxidative stress is the intracellular overproduction of reactive oxygen species (ROS), such as superoxide (O_2_^•−^) and hydroxyl radicals (^•^OH). Nitrosative stress is a related process, involving the overproduction of reactive nitrogen species (RNS), such as nitric oxide (NO^•^) [1,2]. Unlike free radicals, which are chemicals with one or more unpaired electrons, other compounds, such as hydrogen peroxide (H_2_O_2_) and peroxynitrite (ONOO^−^), are non-radicals, but are still capable of causing extensive cellular damage.

The investigation of ROS and RNS, and strategies to block their cellular effects, or their generation in the first place, has been the focus of many reviews and books to date [1,2,3,4]. All cells—whether *in vitro* or *in vivo*—which utilize oxygen for aerobic respiration must maintain a delicate homeostasis between the generation and the subsequent removal of ROS and RNS. Normal, healthy cells are capable of performing adequate reduction/oxidation (redox) homeostasis. Neuronal cells in particular, however, have been found to be vulnerable to oxidative stress, partly due to their intense levels of oxidative respiration. Multiple forms of neural tissue can experience long-term degeneration, including retinal tissue in conditions such as age-related macular degeneration and diabetic retinopathy. Retinal tissue consumes a lot of oxygen, and thus the production of ROS through cellular respiration is also quite high [5]. In addition, this tissue is high in fatty acids, a common target of ROS. Therefore, there is considerable interest in antioxidant treatment for delaying or preventing retinal neurodegeneration. Most data surrounding the effects of oxidative stress in the nervous system, therefore, have focused on finding treatments that will delay or prevent neuronal cell loss rather than other cell types.

**Figure 1:**
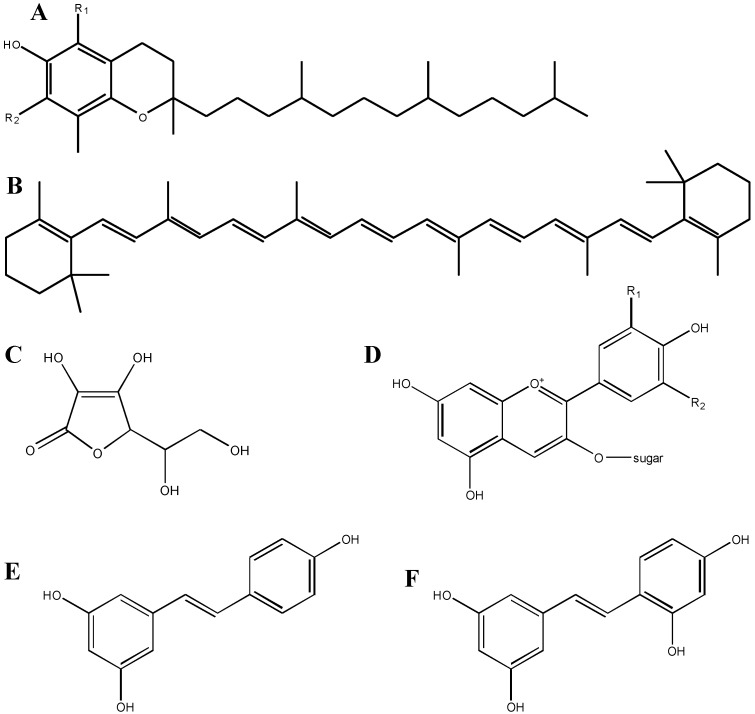
Structures of various antioxidant compounds. **A**: vitamin E (tocopherol); constituents at the R_1_ and R_2_ positions are either –H or –CH_3_; **B**: beta-carotene, a member of the carotenoid family; **C**: vitamin C (ascorbic acid); **D**: anthocyanin; constituents at the R_1_ and R_2_ positions are –H, –OH or –OCH_3_, and the sugar is glucose, galactose or arabinose; **E**: resveratrol; **F**: oxyresveratrol; note the additional OH group as compared to resveratrol.

One of the major tenets of oxidative stress is that endogenous cellular detoxification systems can only handle a finite ROS/RNS load. Because oxidative stress has been linked to many neuropathological and neuropsychiatric conditions, many compounds have been developed that attempt to reduce the cellular load of ROS or RNS [1,6]. Of course, ROS and RNS are normally produced during cellular respiration, and cells have developed endogenous mechanisms of neutralizing these compounds. These endogenous systems are either enzymatic, such as superoxide dismutase (SOD), catalase or glutathione peroxidase, or non-enzymatic, such as the tripeptide glutathione. Because many of the endogenous systems are enzymatic, increasing the amount of intracellular SOD, for example, requires either delayed SOD degradation, or enhanced SOD gene expression and subsequent increased SOD protein production. A potentially easier route could be the addition of exogenous compounds in the diet, the so-called “free radical scavengers” which are, in essence, antioxidants because of their ability to return cells to their normal redox homeostasis. This class of compounds includes commonly known antioxidants, such as vitamin E (tocopherol), vitamin C (ascorbic acid), carotenoids, and polyphenols (Figure 1) [7,8].

These types of dietary molecules are often referred to as nutraceuticals, which are compounds believed to exert a positive effect on health. Plant-derived polyphenols in particular have received a lot of attention for their potential health benefits. For example, the high quantities of polyphenolic compounds found in some species of berries (e.g., *Vaccinium* species such as blueberries and lingonberries), and their reported antioxidant properties, may be beneficial for neurological disorders and processes, such as brain aging [9,10]. Given the idea that increased oxidative stress may be a major contributor to several neurological diseases and brain aging, the ingestion of berries or dietary supplements containing their constituents may have a positive effect on brain health [1,10]. The potential neuroprotective effects of berry-derived polyphenols are discussed in more detail in Section 4.

### 2.2. Structure-Function Relationships of Antioxidant Compounds

The number of people affected by neurological and neurodegenerative diseases continues to rise, which has led to an increased need for new antioxidant, neuroprotective compounds. Much literature has been dedicated to unraveling the mechanisms behind neuroprotective compounds, with the aim of reducing or prohibiting neural loss [11,12]. Like most drug discovery methods, this is a black-box process, in which a definitive cause-effect relationship must be obtained for proof-of-concept. In addition, understanding the relationship between a compound’s structure and its cellular function allows for top-down drug design to improve bioavailability, increase efficacy and reduce toxicity. In general, the efficacy of many of these compounds derives from their structure, and, as seen in Figure 1, antioxidant compounds often have structural similarities that enable them to trap excess electrons from oxidizing compounds. Interestingly, these compounds themselves are capable of being oxidized, and thus require their own neutralization pathways.

The fact that molecules, such as polyphenols, are purported to exert their antioxidant capability by neutralizing unpaired electrons, is given credence by comparing two similar chemicals: resveratrol and oxyresveratrol (Figure 1). Oxyresveratrol is a compound very similar to resveratrol, an antioxidant polyphenol found in the seeds and skins of red grapes. These molecules are identical, other than the addition of another –OH substituent on one of the phenol rings in oxyresveratrol; this additional group may make this compound a stronger antioxidant than resveratrol, in that it can more easily neutralize unpaired electrons. As we describe later, however, this explanation may be too simple, and may downplay the complexity of compounds in brain tissue. For example, one compound that failed to provide reproducible results in two clinical trials for patients with acute ischemic stroke (NXY-059; chemical name: disodium 2,4-disulfophenyl-N-tert-butylnitrone) did not demonstrate protective effects against H_2_O_2_-induced oxidative stress in neuroblastoma cells *in vitro*. However, this compound may have exerted its protective effects by acting as an NO• mimetic [1,13].

Interestingly, one of the reported misconceptions about antioxidants is that, since they perform the same basic function, they are all the same and thus interchangeable [14]. However, that is clearly not the case. For example, an appropriate antioxidant for retinal tissue could preferentially target free radicals and ROS that damage fatty acids due to the high amount of these chemical compounds in this type of tissue. Many research teams believe that anthocyanins found in fruits, such as blueberries and bilberries, could be the answer for protecting this tissue from oxidative stress.

## 3. Screening of Antioxidants Using *in Vitro* Approaches

### 3.1. Cell Lines versus Primary Cultures

Determination of compound efficacy-before the initiation of animal experiments *in vivo* or human clinical trials-requires extensive testing *in vitro* to establish critical parameters, such as toxicity. In many situations, *in vitro* testing is used as a high-throughput screening method for compounds developed via bottom-up molecular synthesis. This type of screening is fast, inexpensive and relatively reliable. Drug screening *in vitro*, in which confounding variables can be controlled for, and cause-effect relationships can be more easily discerned, are often both useful and necessary for elucidating cellular and/or mechanistic effects of compounds. 

The use of cell lines and/or primary cell cultures to devise and test neuroprotective compounds is a widely accepted strategy, and one that is necessary for various regulatory bodies. Cell lines are cells that have become “immortalized”, usually through mutation, and continue to divide, as long as their growth and nutritional requirements are met [15,16]. Many mammalian cell lines are derived from biopsies of cancerous tissue, and often long outlive their original host. Two of the main advantages of using cell lines are their ease of use, and their low cost. A particular cell line can be purchased and maintained almost indefinitely in-house, and requires only a basic cell culture facility. Since the cells have already been removed from their host by the production facility, dissections are not required, and researchers do not require direct access to an animal facility. In addition, the number of human-derived cell lines currently available is extremely diverse, making it easy to compare the effects of one antioxidant compound on a variety of tissue types, including neuronal and glial tissue. In our opinion, this is one of the main strengths of using cell lines for mechanistic studies, as a particular compound may not be efficacious in the brain, but may instead exert its effects on other tissue types.

Of course, cell lines have distinct disadvantages when used to study the antioxidant capability of various compounds. The fact that many cell lines are cancerous makes it difficult to study the effects of compounds on “normal” tissue. Additionally, cell lines are notorious for becoming contaminated with other cancerous cell types: for example, it has been estimated that 29% of cell line stocks are contaminated with HeLa cells [17]. These issues can be avoided when using primary cell cultures, which are derived directly from the host animal through dissection. In the case of experiments analyzing neuroprotection *in vitro*, these cultures are typically derived from embryonic or neonatal brains of mice or rats [18,19,20]. However, these cultures are accompanied by their own specific set of disadvantages when it comes to their use for screening potential antioxidant compounds. Specifically, establishing primary cultures are more expensive, more heavily regulated, requires an animal care facility, animal care protocols, more time, and continuous maintenance.

### 3.2. Rationale for in Vitro Testing of Antioxidants

Cells derived from immortal cell lines or from primary sources are cultured either in suspension (where cells are freely floating in media) or as adherent monolayers on a substrate (usually plastic or glass). The bulk of our experimental work has used adherent cells, and their ability to form a monolayer makes it very easy to visualize cells under a microscope, to perform immunocytochemistry, and so on [18,19,20]. Our aim in this review is to indicate various caveats one should consider with regards to demonstrating neuroprotective compound efficacy *in vitro*. Indeed, we believe that *in vitro* screening can be viewed as a very reliable technique as long as the correct approach is used. The beautiful complexity of the brain, however, could be its own downfall, with regards to *in vitro* testing, as cultures do not maintain the exact cellular architecture of an intact brain, making it difficult to appropriately and accurately extrapolate findings from *in vitro* experiments to the more complex scenario of neuroprotection *in vivo*. The use of isolated cells *in vitro* is a necessary stepping-stone to demonstrating proof-of-concept for neuroprotective compounds. Various studies in our own laboratories have been conducted solely *in vitro*, with recommendations that compounds be further tested *in vivo* [18,19].

Most *in vitro* experiments, using adherent cells, apply compounds (in various doses) directly to cells, in order to generate a dose-response relationship. The assumptions made here are that the negative control treatment will not exert any effects, that there will be an effective dose, and that at high enough concentrations, the compound will kill cells. Of course, these high concentrations do provide critical information about toxicity, but the fear is that the effective dose is higher than what would ever cross the blood-brain barrier (BBB). However, the BBB is often damaged or weakened after various insults, such as stroke or traumatic brain injury, which could allow compounds to cross more easily, and at higher concentrations than in people with normal, undamaged brains. For example, oxyresveratrol was unable to effectively cross the BBB in healthy animals, but was found in much higher concentrations after experimental stroke [21]. A similar rationale was proposed for NXY-059, in that this compound is water-soluble and does not easily cross an intact BBB [1,22]. In this way, *in vitro* experimentation using adherent cells could replicate the situation of compounds crossing the BBB, and provides a rational explanation for continuing to test compound efficacy in this manner.

There is a second major issue that must be considered when gathering data about a compound *in vitro*. The direct application of compounds to cells (including, but not limited to neurons and glia) eliminates any of the changes that are made to those compounds through standard xenobiotic metabolism. In this way, the critical issue of bioavailability is improperly addressed, as unmetabolized antioxidant compounds could likely not reach the brain at the concentration, or in the time-window, required for effective treatment. Again though, a damaged or weakened BBB may assist in timely delivery to neural tissue. It is also possible that metabolites of various compounds may have antioxidant activity as well, which would not typically be captured with *in vitro* approaches. In the last sections of this review, we focus on the use of compounds administered *in vivo*, and how we can use these methodological caveats to improve our findings *in vitro*. In particular, we examine some positive evidence for neuroprotection *in vivo* using berry-derived polyphenolic compounds.

## 4. *In Vivo* Studies with Antioxidants

### 4.1. Berry-Derived Polyphenolics

The potential of various berries to protect the brain from aging and neurodegenerative disorders has gained increased attention in recent years, in large part due to their high polyphenol content and antioxidant capacity [9,10]. Many studies using whole animals, usually rodents, have been conducted recently in order to evaluate the effects of berries on the nervous system. For example, dietary supplementation with high amounts of blueberries can decrease age-related behavioral deficits in rats [23]. In a recent study conducted using a mouse model of Alzheimer’s disease, treatment with bilberries, which are rich in polyphenols, decreased the extent of behavioral abnormalities associated with the disease [24]. Another study demonstrated that rats fed a diet enriched with blueberries can protect the brain against oxidative stress and associated learning deficits [25]. Perhaps most surprisingly, diets enriched with blueberries over the course of several weeks have been shown to protect animals from damage induced by insults as severe as ischemic stroke [26,27].

Some *in vivo* studies have specifically investigated the protective effects of polyphenols against retinal damage. In one such study mice were treated prophylactically with bilberry extract, which was quantified and found to contain about 39% anthocyanins, after which they were subjected to retinal inflammation [28]. The findings suggested that the high levels of anthocyanins in bilberry extract were able to directly scavenge ROS produced via inflammation, and provide substantial neuroprotection to retinal tissue. In another study, rats were fed blueberries by oral gavage prophylactically for two or seven weeks before being subjected to light-induced retinopathy, and both groups experienced significant retinal neuroprotection [29].

These types of *in vivo* experimental approaches offer several advantages over cell-based models. For example, *in vivo* studies are more realistic in the sense that animals must ingest a diet containing berries or their extracts, as would humans. Animals can also be fed for various periods of time (e.g., a single day *versus* many weeks), and with different percentages of berries constituting the diet. Several different behavioral tests can be administered in animals to measure motor and/or cognitive functions, and the results can be compared to animals treated with a non-berry enriched diet. In addition, at the end of behavioral testing, several other analyses can be conducted, such as immunocytochemical and histological analysis of various brain areas and other parts of the body, including the liver, heart, and kidneys. Additionally, as mentioned above, the effects of various berries or extracts can be tested in genetic rodent models for various diseases, such as Alzheimer’s disease [24].

Despite the many inherent advantages of *in vivo* approaches, there are also some drawbacks. These types of experiments can be financially expensive, especially when considering the cost of maintenance of many animals over the course of several weeks or months. *In vivo* experimentation is not a method that can easily be justified for compound screening, due to the cost, time, and number of animals needed to observe an effect. For this reason, *in vitro* tests must be rigorous and well defined, so that promising compounds are then explored *in vivo*. As with *in vitro* approaches, animals often receive a high amount of berries or extracts in their diets that may not be realistically achieved with humans in order to see a definitive effect. Lastly, many studies do not analyze the extent to which berry-derived polyphenols have entered the brain, making it difficult to determine a mechanism by which a positive effect may have occurred.

### 4.2. Polyphenolic Bioavailability

There is now substantial evidence suggesting that the ingestion of diets high in berries can have positive effects on the brain, not just in rodents, but also in the human population [30,31]. However, the data remain inconclusive as to whether this is due to direct or indirect effects on nervous system tissue. Some recent research has demonstrated that dietary polyphenols can cross the BBB [30], and anthocyanin compounds specifically have been detected in brain tissue after oral administration to rodents [32,33,34] as well as pigs [35,36]. Some estimates of specific anthocyanins in brain tissue are in the sub-nanomolar range (0.2–0.25 nmol/g tissue) [33,34], whereas some others are as low as the femtomolar range [36]. Although it cannot be expected that every research group conducting *in vitro* experiments on brain cells, or other *in vivo* tests, measure polyphenol levels in brain tissue of berry-fed animals, it is important to utilize data from bioavailability studies in order to test concentrations of antioxidant compounds that would reach the brain. In some of the previous *in vitro* work that we have conducted, we found that the final concentration of blueberry and lingonberry extracts that we tested in cell cultures was 0.833 μg/ml of fruit extract and 0.083 μg/ml of leaf extract [19]. In other previous work we conducted chemical analysis of commercially available lingonberry extracts and found that these extracts contain an estimated 63.7 mg of cyanidin-3-galactoside per 100 mg of fresh extract weight (unpublished data). If our fresh lingonberry extracts tested *in vitro* contained a similar amount of this compound, this would translate to the cultured cells being exposed to approximately a 10 nM concentration of fruit extract and 1 nM in leaf extract. Talavera *et al.* [33] detected a level of another cyanidin compound (cyanidin-3-glucoside) of 0.25 nmol equivalent per g of tissue. Therefore, the amount of extract that we tested for neuroprotective effects in cultures is most likely somewhat higher than what might be achieved in the brain after oral administration. The amount we added to cultures is also much higher than femtomolar estimates in pigs that had ingested polyphenols orally [36]. However, the polyphenol measurements occurred 18 h postprandial in these latter studies, so it is possible that higher polyphenol levels may have been detected in the brain if measurements had occurred earlier. Most studies in whole animals also feed animals berry-rich diets for several weeks. However, the extent to which berry-derived polyphenols enter the brain from short periods of ingestion (e.g., a day or a week), or how long these constituents stay present in the brain, is not known.

A recent review highlighted ten common misconceptions about antioxidants, including the purported ability of these compounds to cure any disease, or to increase one’s lifespan [14]. In addition, the authors point out that a “true” antioxidant should be efficacious at its target (e.g., DNA) at relatively low concentrations, repudiating the notion that “the more antioxidant, the better” when it comes to administration. In relation to this, some researchers have suggested that tissue storage of anthocyanins may become saturated after several weeks (four to eight) of supplementation in the diet [37], which would therefore limit availability to the brain. Recently, it was demonstrated that tissue saturation with antioxidants need not occur in order to provide a neuroprotective effect [29]. We feel that this is a critical finding, in that low compound bioavailability may still provide marked neuroprotection.

Another important consideration in study design using berries and their constituents is that polyphenolic compounds contained in extracts that are tested *in vitro* may not be the predominate forms that would actually enter the brain. In fact, some recent studies have found that although anthocyanins have a fairly high bioavailability, they also undergo significant metabolism, producing diverse metabolites [38,39]. Some experimental evidence suggests that certain polyphenolic compounds are maintained in their natural glycosylated form [32,33]. Xenobiotic metabolism also most likely contributes to the amounts and different forms of polyphenols that cross the BBB, as additional evidence has demonstrated that glucuronide forms of anthocyanins can be detected in the brain [36]. A significant amount of additional research is needed in order to determine the specific types of polyphenolic compounds that can enter the brain, and to what extent.

## 5. Other Potential Mechanisms of Protection of Natural Compounds

Due to the extensive amount of information suggesting that oxidative stress can contribute to a variety of disease states, including neurological disorders, the ability of polyphenolic compounds to act as antioxidants has arguably received the most attention as far as their mechanism of protection. Although polyphenols such as anthocyanins and flavonols may exert some of their neuroprotective effects by acting directly as antioxidants, they likely have other important effects, such as scavenging reactive nitrogen species, activating protective cell signaling pathways, or altering the expression of various proteins [30,31,40,41]. This suggestion is strengthened by evidence that the bioavailability of these compounds in the brain is much lower than the levels of endogenous antioxidant compounds [41]. The compound resveratrol was initially believed to exert positive health benefits by acting as an antioxidant compound. However, recently it has been found that resveratrol exhibits its potential anti-aging effects by acting primarily as a phosphodiesterase inhibitor [42]. Although oxyresveratrol has indeed been shown to exert antioxidant effects [43], due to its similarity in structure to resveratrol, it is possible that it also has varied mechanisms of action resulting in neuroprotection.

One possibility is that polyphenols exert beneficial properties through purported hormetic effects. One example of such a mechanism is that low levels of these compounds appear to activate the transcription factor Nrf2, which can induce the production of endogenous antioxidant enzymes and other related protective compounds [30,41]. Polyphenolic compounds can also decrease the level of pro-inflammatory mediators in the brain, such as tumor necrosis factor-α and a variety of interleukins [31]. The anti-nitrosative properties of polyphenols may also contribute significantly to protecting the brain from damage compared to antioxidant effects, by scavenging excessive damaging compounds, such as peroxynitrite. It can also not be ruled out that polyphenols have an indirect effect on the brain that leads to beneficial effects. For example, antioxidant effects could be positive for the cardiovascular system, which may lead to an increase in blood flow to the brain. This secondary effect could improve cognitive function both in experimental animals and in humans. These latter biological effects of polyphenols in the brain are poorly understood and warrant further investigation.

## 6. Conclusions

Oxidative stress is believed to contribute to a wide variety of nervous system disorders, including neurodegenerative diseases, brain aging, and insults such as traumatic brain injury and stroke. Therefore, antioxidant compounds have received significant attention as potential treatments for a variety of brain abnormalities, and evidence suggests that antioxidant compounds can be beneficial for the nervous system. Assessment of antioxidant compounds is typically conducted using a variety of *in vitro* and *in vivo* techniques. In this review, we have illustrated many of the advantages and disadvantages of both approaches. For example, although *in vitro* cell culture systems are quite useful for screening antioxidant compounds for neuroprotective potential, cell lines and cells directly isolated from brain tissue do not maintain the exact properties of an intact brain. Although it is generally acceptable to add a high concentration of a compound in order to determine if there are any potential protective effects, the level that an antioxidant compound may achieve in the brain is impossible to determine without *in vivo* animal studies. More studies are currently needed to determine the bioavailability of compounds with high antioxidant capacity, such as polyphenols or similar substances. Although these latter types of studies primarily need to be conducted using *in vivo* experimentation, complimentary *in vitro* studies will continue to significantly aid in deciphering the cellular mechanisms by which antioxidants are truly beneficial, as neuroprotection may be mediated through other mechanisms that are unrelated to alleviating or preventing oxidative stress. At this point however, it is sufficient to say that, although *in vitro* studies provide a wealth of information on various intrinsic compound parameters, we feel that it is highly likely that many compounds incorrectly enter animal trials, with no subsequent success *in vivo*, and that many putative neuroprotective compounds never enter animal trials because of the means used to evaluate their potential protective efficacy *in vitro*. A careful use of both *in vitro* and *in vivo* approaches is necessary in order to lead to the development of antioxidant therapeutic compounds for the human population.
